# Haemodynamic implications of cardiovascular magnetic resonance pulmonary capillary wedge pressure in acute myocardial infarction

**DOI:** 10.1093/ehjimp/qyaf086

**Published:** 2025-07-25

**Authors:** Pankaj Garg, Aradhai Bana, Gareth Matthews, Tiya Bali, Rui Li, Zia Mehmood, Liang Zhong, Rob J van der Geest, Sven Plein, John P Greenwood, Peter Swoboda

**Affiliations:** The Bob Champion Research & Education Building, Rosalind Franklin Road, University of East Anglia, Norwich NR4 7UQ, UK; Cardiology Department, Colney Lane, Norfolk and Norwich University Teaching Hospitals, Norwich NR4 7UY, UK; The Bob Champion Research & Education Building, Rosalind Franklin Road, University of East Anglia, Norwich NR4 7UQ, UK; The Bob Champion Research & Education Building, Rosalind Franklin Road, University of East Anglia, Norwich NR4 7UQ, UK; Cardiology Department, Colney Lane, Norfolk and Norwich University Teaching Hospitals, Norwich NR4 7UY, UK; The Bob Champion Research & Education Building, Rosalind Franklin Road, University of East Anglia, Norwich NR4 7UQ, UK; The Bob Champion Research & Education Building, Rosalind Franklin Road, University of East Anglia, Norwich NR4 7UQ, UK; Cardiology Department, Colney Lane, Norfolk and Norwich University Teaching Hospitals, Norwich NR4 7UY, UK; The Bob Champion Research & Education Building, Rosalind Franklin Road, University of East Anglia, Norwich NR4 7UQ, UK; Cardiology Department, Colney Lane, Norfolk and Norwich University Teaching Hospitals, Norwich NR4 7UY, UK; SingHealth Building, Outram Road, National Heart Centre Singapore 169608, Singapore; Division of Image Processing, Leiden University Medical Center, Leiden, The Netherlands; The Institute of Cardiovascular and Metabolic Medicine, University of Leeds, Woodhouse Lane, Leeds LS2 9JT, UK; The Institute of Cardiovascular and Metabolic Medicine, University of Leeds, Woodhouse Lane, Leeds LS2 9JT, UK; Baker Heart and Diabetes Institute, 99 Commercial Rd, Melbourne VIC 3004, Australia; The Institute of Cardiovascular and Metabolic Medicine, University of Leeds, Woodhouse Lane, Leeds LS2 9JT, UK

**Keywords:** humans, ST elevation myocardial infarction, prognosis, follow-up studies, heart failure, left ventricular dysfunction, heart atria

## Abstract

**Aims:**

Cardiovascular magnetic resonance (CMR)-derived pulmonary capillary wedge pressure (PCWP) has demonstrated diagnostic and prognostic utility in heart failure patients. However, its clinical value in acute myocardial infarction (AMI) remains undetermined. This study investigates the relationship between CMR-derived PCWP, myocardial injury, and left ventricular (LV) remodelling in re-perfused acute ST-elevation myocardial infarction (STEMI).

**Methods and results:**

Sixty-nine patients with STEMI underwent CMR within 48 h and at 3 months. PCWP was estimated using the sex-specific equation: CMR PCWP: 5.7591 + (0.07505 × left atrial volume) [0.05289 × left ventricular mass (LVM)] − (1.9927 × sex) [female = 0; male = 1], where LAV is left atrial volume (mL) and LVM is left ventricular mass (g). LV remodelling was assessed via changes in LV end-diastolic volume (LVEDV) and ejection fraction (LVEF). Patients with high CMR PCWP (≥18 mmHg) exhibited greater myocardial scar burden (28.5% vs. 17.2%, *P* = 0.0008) and microvascular obstruction (7.6% vs. 2.5%, *P* < 0.0001). They also had higher acute LVEDV (193.7 ± 39.7 vs. 158.0 ± 29.5 mL, *P* < 0.0001) and lower LVEF (41.4 ± 10.4% vs. 48.5 ± 9.2%, *P* = 0.0066). At follow-up, higher baseline CMR PCWP was associated with greater LV remodelling (*P* < 0.0001) and persistently reduced LVEF (45.4 ± 10.2% vs. 55.0 ± 10.3%, *P* = 0.0005). Regression analysis confirmed baseline PCWP as an independent predictor of follow-up LVEF (*P* = 0.0036).

**Conclusion:**

CMR-derived PCWP may be a valuable biomarker in STEMI, identifying patients at risk of adverse remodelling and LV dysfunction. Its integration into clinical practice may enhance risk stratification and guide targeted therapies.

## Teaser

CMR-modelled PCWP has haemodynamic implications in acute MI.

## Introduction

Acute ST-elevation myocardial infarction (STEMI) remains a leading cause of cardiovascular morbidity and mortality worldwide. A critical determinant of prognosis in STEMI is left ventricular (LV) filling pressure, often assessed as pulmonary capillary wedge pressure (PCWP). Elevated PCWP reflects impaired relaxation, increased ventricular stiffness, and heightened myocardial oxygen demand—all of which contribute to adverse remodelling and higher short- and long-term mortality.^1,2^ Mechanistically, diastolic dysfunction in the infarcted myocardium exacerbates pulmonary congestion and heart failure risk, while elevated PCWP also impairs coronary microcirculation, further compromising myocardial perfusion.^3^ Clinically, recognition of elevated LV filling pressure is essential for guiding therapeutic interventions—such as nitrates or other preload-reducing agents—that can mitigate ventricular stress and potentially improve outcomes.^4^

Cardiovascular magnetic resonance (CMR) has emerged as a powerful imaging modality for the comprehensive assessment of acute myocardial infarction (AMI), providing precise quantification of volumes, function, and infarct size. More recently, CMR-derived techniques have been developed to estimate PCWP—a surrogate measure that closely reflects LV filling pressure. This non-invasive method has been demonstrated to have prognostic value, as elevated CMR-derived PCWP is associated with an increased risk of mortality in patients with heart failure.^5^ Additionally, CMR-derived PCWP during exercise stress testing has shown good diagnostic accuracy for detecting heart failure with preserved ejection fraction (HFpEF) and can predict hospitalization within 24 months.^6^ Even at the population level, data from the UK Biobank indicate that CMR-derived PCWP is independently associated with new-onset heart failure.^7^ However, the specific haemodynamic and clinical impact of CMR-derived PCWP in AMI has yet to be fully characterized. Understanding this relationship could refine risk stratification and guide targeted therapy, underscoring the urgent need for further investigation into the haemodynamic consequences of CMR PCWP.

In this study, we hypothesized that PCWP derived from CMR would exhibit a haemodynamic correlation with markers of congestion and functional impairment and would be able to predict ventricular remodelling following re-perfused ST-elevation myocardial infarction (STEMI). Consequently, the primary objective of this study was to evaluate the relationship between CMR-derived PCWP, acute myocardial injury, and subsequent LV remodelling and function during the convalescent phase.

## Methods

### Study population

This prospective cohort study recruited patients diagnosed with acute re-perfused STEMI and this cohort has been described previously.^8,9^ Patient recruitment was conducted at Leeds Teaching Hospitals NHS Trust (Leeds, UK). As part of routine clinical care at the initial medical contact, all participants received pre-treatment with acetylsalicylic acid, a P2Y12 inhibitor, and heparin, following current STEMI guidelines.^10^ Eligible patients were in sinus rhythm and underwent CMR imaging within 48 h of the index event and again at a 3-month follow-up. Exclusion criteria comprised a history of myocardial infarction or prior coronary revascularization, non-ischaemic cardiomyopathy, significant renal impairment (estimated glomerular filtration rate < 30 mL/min/kg), haemodynamic instability, and any contraindications to CMR imaging. Additionally, patients with valvular disease (ranging from mild to severe) were excluded to avoid confounding intra-cardiac flow disturbances.

### Study ethics and regulations

This study adhered to the ethical principles outlined in the 1964 Declaration of Helsinki and its subsequent amendments. Ethical approval for data collection and management was granted by the National Research Ethics Service of the United Kingdom (Reference: 12/YH/0169). Informed written consent was obtained from all study participants.

### CMR protocol and analysis

CMR imaging was performed using a 1.5 Tesla (T) scanner (Ingenia, Philips, Best, The Netherlands) equipped with a 28-channel phased-array cardiac receiver coil. The imaging protocol included cine imaging and late gadolinium enhancement (LGE) imaging. Cine imaging was conducted using an electrocardiogram (ECG)-gated, balanced steady-state free precession sequence obtained in the two-chamber, three-chamber, and four-chamber views alongside a contiguous short-axis cine stack covering the entire LV. Each cine acquisition was performed in a single breath-hold at mild expiration. LV volumes, mass, and ejection fraction were calculated from the short-axis cine stack.

LGE imaging was conducted 10–15 min post-administration of a gadolinium-based contrast agent (Dotarem, Guerbet; 0.2 mmol/kg), utilizing a T1-weighted segmented inversion-recovery gradient-echo sequence in the same short-axis orientation as the cine images. Infarct size was determined using the full-width at half-maximum (FWHM) technique, which involves identifying the peak signal intensity within the hyper-enhanced area and applying a threshold at 50% of this maximum to delineate the infarcted tissue. This method has been shown to provide reproducible and accurate quantification of myocardial scar. Microvascular obstruction (MVO) was identified on LGE images as hypo-enhanced regions within the hyper-enhanced infarcted myocardium. The extent of MVO was quantified by manual planimetry on each relevant short-axis slice, summing the areas of hypoenhancement and expressing the total volume as a percentage of LV mass. This approach allows for the assessment of the impact of MVO on post-infarction remodelling and patient prognosis.

### LV remodelling and functional changes

Given the absence of a universally accepted definition of LV remodelling, LV remodelling over the 3-month follow-up period was assessed by measuring absolute changes in LV end-diastolic volume (LVEDV) from baseline to follow-up. Measuring absolute change in LV end-diastolic volume allows for a nuanced analysis of the relationships between remodelling and clinical outcomes. A similar approach was used for LV ejection fraction. Importantly, we also planned to do relative delta-change analysis of LV volumes.

### Estimation of left ventricular filling pressure using sex-specific CMR-derived equations

To estimate left ventricular filling pressure (LVFP), we utilized a sex-specific equation derived from CMR metrics, as described previously. This equation incorporates left atrial volume (LAV) and left ventricular mass (LVM) to calculate the pulmonary capillary wedge pressure (PCWP), serving as a surrogate for LVFP.^11^ LAV was measured using the biplane area-length method from the 2two and four-chamber cine images at end-systole. Left ventricular mass was determined through short-axis segmentation in end-diastole using established techniques. Papillary muscles were included in blood volume. These parameters were integrated into sex-specific equations to derive estimates of PCWP, providing a robust method for evaluating diastolic function. The sex-specific equation is as follows:


CMRPCWP=5.7591+(0.07505*LAV)+(0.05289*LVM)–(1.9927*sex)[female=0;male=1]

where PCWP is the pulmonary capillary wedge pressure (mmHg), LAV is the left atrial volume (mL), and LVM is the left ventricular mass (g).

All CMR image analyses were conducted using dedicated research software (MASS version 2021-Exp, Leiden University Medical Center, Leiden, The Netherlands). CMR contour tracings, including volume/function assessments and LGE segmentation, were performed by R.J.G at core-lab (Leiden). All CMR analyses were conducted in a blinded fashion at an independent core lab, with analysts masked to both clinical data and group allocation to minimize bias.

### Statistical analysis

Normality testing was carried out for all continuous variables individually using the Kolmogorov–Smirnov Test (see Supplementary data online, *Table S1*). Most of the continuous variables demonstrated normality, so parametric statistical methods were employed in the current study. This decision was supported by prior evidence demonstrating that the *t*-test and related parametric methods remain robust under moderate deviations from normality.^12^ Since observed values did not show extreme skewness or kurtosis, this approach was deemed reasonable and efficient for detecting intergroup differences. All continuous variables were expressed as mean ± standard deviation.

### Sample size determination

For the primary endpoint of follow-up left ventricular ejection fraction (LVEF) differences between patients with PCWP < 18 mmHg and those with PCWP ≥ 18 mmHg, we assumed an intergroup difference of 9.6% with an estimated standard deviation of 9%, yielding an effect size of ∼1.07.^13–15^ Based on standard parameters for a two-sided test with an alpha of 0.05% and 80% power, the minimum required sample size was estimated to be ∼14 patients per group.

### Data analysis and regression modelling

Group comparisons for continuous variables (e.g. volumetric indices, LVEF, laboratory measures) were carried out using two-sample *t*-tests, while categorical variables (e.g. sex, hypercholesterolaemia, medication use) were compared with Pearson *χ*^2^ tests. All significance levels were set at *P* < 0.05. A stepwise multiple linear regression model was employed to identify independent predictors of follow-up LVEF at 3 months, considering baseline LVEF, baseline PCWP, MVO, age, sex, and scar burden as candidate variables. Predictors were retained in the final model only if they satisfied *P* < 0.05. Diagnostic plots of residuals confirmed linearity, normality, and homoscedasticity. A conventional variance inflation factor threshold of <5 was adopted for ruling out harmful multicollinearity. The adjusted *R*^2^ and overall *F*-test assessed model performance. Statistical analyses were conducted with IBM SPSS Statistics (Version 29) and MedCalc (Version 23.1.7).

## Results

### Study population

A total of 69 patients were included, of whom 46 (67%) had a CMR-derived PCWP < 18 mmHg and 23 (33%) had a PCWP ≥ 18 mmHg (*Table 1*). Overall, mean age was comparable between groups (about 61 years). However, the higher-PCWP group consisted entirely of men, showed significantly larger body surface area (2.1 ± 0.1 vs. 1.9 ± 0.2 m2, *P* < 0.0001), prolonged QRS duration (107.5 ± 22.7 vs. 91.9 ± 23.2 ms, *P* = 0.0456), and elevated serum urea levels (6.9 ± 2.5 vs. 5.6 ± 1.5 mmol/L, *P* = 0.0109). In contrast, the two cohorts had similar key clinical parameters, such as systolic blood pressure, heart rate, and multi-vessel disease burden. Medication use was high in both groups, although angiotensin-converting enzyme inhibitors, beta-blockers, and aspirin each reached statistical significance due to near-universal adoption in the lower-PCWP arm.

**Table 1:** Patient demographics are stratified as per CMR PCWP

	CMR PCWP < 18 mmHg	CMR PCWP ≥ 18 mmHg	*P*-value
Number of patients	46	23	
Age (years)	61.6 ± 11.6	61.8 ± 10.9	0.9614
Body surface area (m^2^)	1.9 ± 0.2	2.1 ± 0.1	<0.0001
Male sex, *n* (%)	35 (76%)	20 (87%)	0.2934
Smoking status, *n* (%)	28 (61%)	10 (43%)	0.1741
Hypertension, *n* (%)	14 (30%)	5 (22%)	0.4492
Hypercholesterolaemia, *n* (%)	10 (22%)	8 (35%)	0.2482
Diabetes mellitus, *n* (%)	7 (15%)	2 (9%)	0.4516
Family history of coronary artery disease, *n* (%)	20 (43%)	9 (39%)	0.7320
Cerebrovascular accident, *n* (%)	2 (4%)	0 (0%)	0.3137
Peripheral vascular disease, *n* (%)	1 (2%)	1 (4%)	0.6145
Heart rate (bpm)	77.6 ± 17.2	80.6 ± 28.2	0.5935
Systolic blood pressure (mmHg)	142.2 ± 37.8	142.7 ± 31.0	0.9579
Killip class I, *n* (%)	40 (87%)	17 (74%)	0.1849
Killip class II, *n* (%)	1 (2%)	2 (9%)	0.1849
Ventricular fibrillation, *n* (%)	1 (2%)	2 (9%)	0.1849
Single-vessel disease, *n* (%)	24 (52%)	15 (65%)	0.5404
Two-vessel disease, *n* (%)	13 (28%)	4 (17%)	0.4262
Three vessel disease, *n* (%)	5 (11%)	4 (17%)	0.4730
Left main stem disease, *n* (%)	2 (4%)	0 (0%)	0.3029
Left anterior descending artery involvement, *n* (%)	21 (46%)	14 (61%)	0.3102
Circumflex artery involvement, *n* (%)	7 (15%)	6 (26%)	0.3208
Right coronary artery involvement, *n* (%)	19 (41%)	8 (35%)	0.5089
QRS duration (ms)	91.9 ± 23.2	107.5 ± 22.7	0.0456
TIMI grade flow pre-intervention			
0	31 (67%)	20 (87%)	0.4053
1	5 (11%)	1 (4%)	
2	3 (7%)	0 (0%)	
3	4 (9%)	2 (9%)	
TIMI grade flow post-intervention			
0	0 (0%)	1 (4%)	0.4617
1	1 (2%)	0 (0%)	
2	3 (7%)	1 (4%)	
3	39 (85%)	21 (91%)	
Red blood cell count (**×**10**⁶**/**µ**L)	138.6 ± 13.1	145.5 ± 14.2	0.0544
White blood cell count (**×**10^3^/**µ**L)	11.3 ± 3.2	12.1 ± 3.3	0.3834
Haematocrit (%)	0.4 ± 0.04	0.4 ± 0.05	0.1193
Sodium (mmol/L)	138.3 ± 2.4	139.1 ± 2.7	0.2275
Potassium (mmol/L)	4.2 ± 0.4	4.3 ± 0.5	0.3829
Urea (mmol/L)	5.6 ± 1.5	6.9 ± 2.5	0.0109
Creatinine (mmol/L)	74.0 ± 17.9	81.2 ± 21.7	0.1535
Estimated glomerular filtration rate (mL/min/1.73 m2)	82.7 ± 12.4	80.9 ± 13.6	0.5873
Creatine kinase (U/L)	1906.9 ± 1303.2	2618.5 ± 2075.0	0.1618
C-reactive protein (mg/L)	19.7 ± 20.1	57.4 ± 49.1	0.0616
Peak troponin (ng/L)	3782.2 ± 17 210.0	44 795.9 ± 12 016.0	0.1265
Haemoglobin A1c (% or mmol/mol)	42.1 ± 11.4	44.2 ± 17.2	0.6247
Angiotensin-converting enzyme inhibitor	43 (93%)	20 (87%)	<0.0001
HMG-CoA reductase inhibitor	40 (87%)	19 (83%)	0.601
Beta-adrenergic receptor blocker	42 (91%)	20 (87%)	<0.0001
Acetylsalicylic acid	43 (93%)	21 (91%)	<0.0001
Ticagrelor or clopidogrel	43 (93%)	19 (83%)	0.196
Mineralocorticoid receptor antagonist	2 (4%)	3 (13%)	0.1797
Anticoagulant therapy	1 (2%)	1 (4%)	0.574
Loop diuretic therapy	1 (2%)	2 (9%)	0.2036

TIMI, thrombolysis in myocardial infarction grade after percutaneous coronary intervention.

### Temporal change in wedge pressure

**Figure 1:**
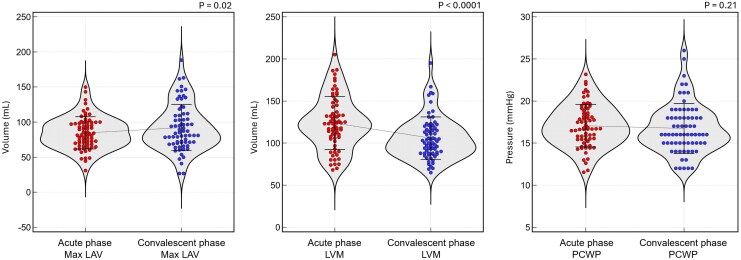
Violin plots demonstrating temporal changes in CMR-derived volumetric parameters and PCWP between acute and convalescent phases post-myocardial infarction. Max LAV after 3-month post-indexed event was markedly larger; in contrast, LVM was significantly lower in the convalescent phase as compared with the acute phase. PCWP showed no statistically significant change between the acute phase and after 3-month post-indexed event. LAV, left atrium volume; LVM, left ventricular mass; PCWP, pulmonary capillary wedge pressure.

PCWP showed no significant change between the convalescent and acute phases (16.74 ± 2.96 mmHg vs. 17.07 ± 2.53 mmHg, *P* = 0.21). LAV were significantly larger after 3 months post-indexed event (92.198 ± 32.97 mL vs. 84.58 ± 22.99 mL, *P* = 0.02), while LVM were significantly lower compared with the acute phase (105.93 ± 25.13 g vs. 123.87 ± 31.31 g, *P* < 0.0001) (*Figure 1*).

### CMR characteristics in patients with high PCWP

Patients in the higher-PCWP group showed more adverse cardiac remodelling on both the acute-phase and 3-month follow-up CMR studies. At the acute phase, patients with PCWP ≥ 18 mmHg had significantly larger left ventricular end-diastolic and end-systolic volumes (193.7 ± 39.7 vs. 158.0 ± 29.5 mL; and 115.7 ± 39.9 vs. 82.6 ± 25.8 mL, respectively; both *P* = 0.0001), lower LVEF (41.4 ± 11.4% vs. 48.5 ± 9.2%, *P* = 0.0066), and greater LVM (149.8 ± 24.4 vs. 110.9 ± 26.0 g, *P* < 0.0001) (*Table 2*). They also demonstrated higher myocardial scar percentage and more pronounced microvascular obstruction.

**Table 2:** CMR characteristics are stratified as per CMR PCWP

	CMR PCWP < 18 mmHg	CMR PCWP ≥ 18 mmHg	*P*-value
Acute phase CMR study (within 48 h)			
Left ventricular end-diastolic volume (mL)	158.0 ± 29.5	193.7 ± 39.7	0.0001
Left ventricular end-systolic volume (mL)	82.6 ± 25.8	115.7 ± 39.9	0.0001
Left ventricular stroke volume (mL)	75.4 ± 15.2	78.0 ± 19.2	0.5383
Left ventricular ejection fraction (%)	48.5 ± 9.2	41.4 ± 11.4	0.0066
Left ventricular end-diastolic mass (g)	110.9 ± 26.0	149.8 ± 24.4	<0.0001
Myocardial scar percentage (%)	17.2 ± 12.1	28.5 ± 13.5	0.0008
Microvascular obstruction (%)	2.5 ± 3.8	7.6 ± 5.9	<0.0001
Mitral regurgitation volume (mL)	3.3 ± 3.1	5.5 ± 3.5	0.0084
Convalescent phase CMR study (at 3 months)			
Left ventricular end-diastolic volume (mL)	159.8 ± 36.6	212.8 ± 61.0	<0.0001
Left ventricular end-systolic volume (mL)	73.8 ± 28.2	121.0 ± 56.3	<0.0001
Left ventricular stroke volume (mL)	86.1 ± 19.7	91.8 ± 14.6	0.2259
Left ventricular ejection fraction (%)	55.0 ± 10.3	45.4 ± 10.2	0.0005
Absolute LV end-diastolic volume remodelling (mL)	1.8 ± 22.2	19.2 ± 39.1	0.02
Delta change in percentage (positive value: reflects increase at convalescent phase)			
Left ventricular end-diastolic volume (Δ%)	−1.2 ± 15.9	6.2 ± 15.5	0.0735
Left ventricular end-systolic volume (Δ%)	−19.1 ± 32.4	−1.6 ± 21.7	0.0227
Left ventricular stroke volume (Δ%)	10.2 ± 16.9	14.0 ± 19.9	0.4043
Left ventricular ejection fraction (Δ%)	10.9 ± 12.5	8.6 ± 15.1	0.5071
Left ventricular end-diastolic mass (Δ%)	−12.0 ± 15.6	−29.2 ± 22.1	0.0004

### Remodelling in convalescent phase

**Figure 2:**
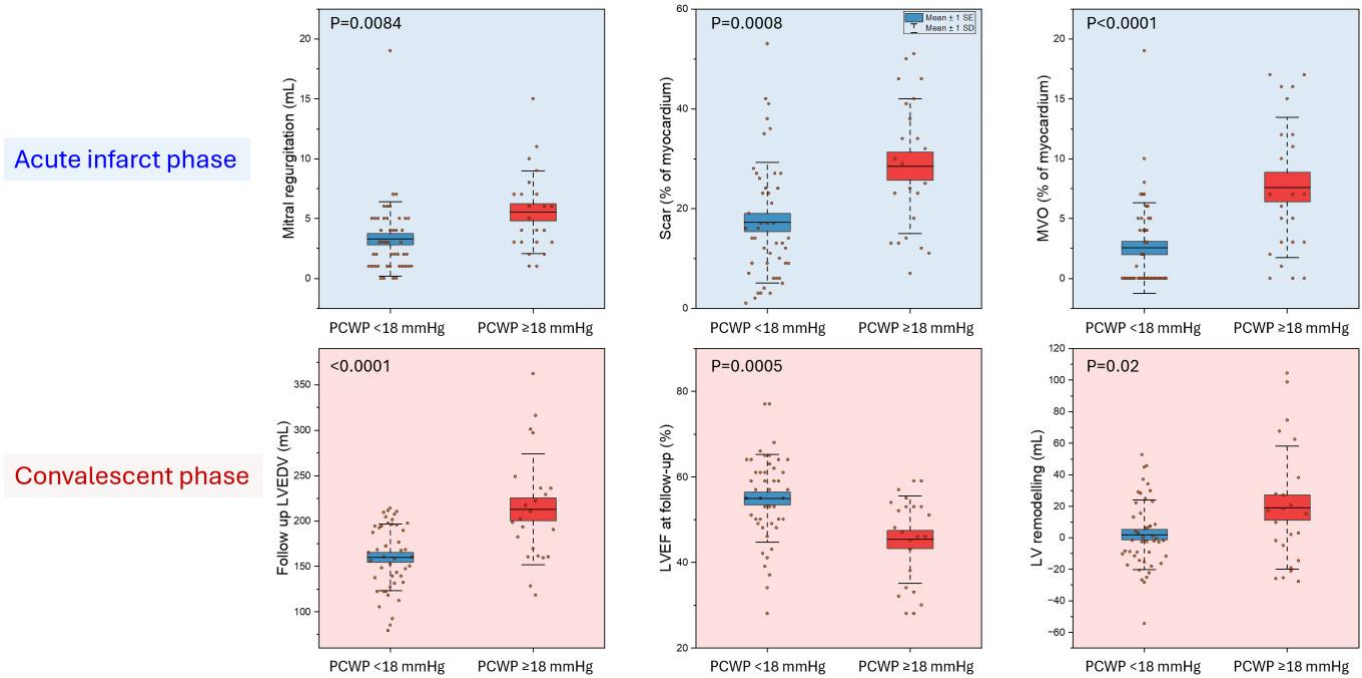
Impact of CMR-derived PCWP on acute infarct characteristics (top row) and long-term cardiac remodelling (bottom row). Patients with elevated PCWP (≥18 mmHg) during the acute infarct phase exhibited higher mitral regurgitation volume, increased myocardial scar burden, and greater microvascular obstruction compared to those with PCWP <18 mmHg. In the convalescent phase, higher baseline CMR-derived PCWP was associated with reduced LV ejection fraction at follow-up, increased LV end-diastolic volume, and greater LV remodelling, indicating a sustained adverse impact of elevated PCWP on myocardial structure and function post-infarction. PCWP, pulmonary capillary wedge pressure; MVO, microvascular obstruction; LV, left ventricular; EF, ejection fraction; EDV, end-diastolic volume.

At convalescent phase, there was significant increase in absolute change in left ventricular end-diastolic volume from acute stage (*Table 2*, *Figure 2*, and Supplementary data online, *Table S2*). However, when looking at relative delta change in percentage, the infarct patients with PCWP < 18 mmHg exhibited a −1.2 ± 15.9% change in LVEDV at convalescence, whereas those with PCWP ≥ 18 mmHg showed a 6.2 ± 15.5% increase (*P* = 0.0735). Left ventricular end-systolic volume decreased by 19.1 ± 32.4% in infarct patients with PCWP ≤ 18 mmHg, compared with a 1.6 ± 21.7% reduction in those with PCWP > 18 mmHg (*P* = 0.0227). Left ventricular stroke volume increased by 10.2 ± 16.9% among infarct patients with PCWP ≤ 18 mmHg and by 14.0 ± 19.9% among those with PCWP > 18 mmHg (*P* = 0.4043). LVEF rose by 10.9 ± 12.5% in infarct patients with PCWP ≤ 18 mmHg vs. 8.6 ± 15.1% in those with PCWP > 18 mmHg (*P* = 0.5071). Finally, LVM declined by 12.0 ± 15.6% in infarct patients with PCWP < 18 mmHg, whereas it declined by 29.2 ± 22.1% in those with PCWP ≥ 18 mmHg (*P* = 0.0004).

### Correlation of CMR-PCWP with clinically relevant outcomes

**Figure 3:**
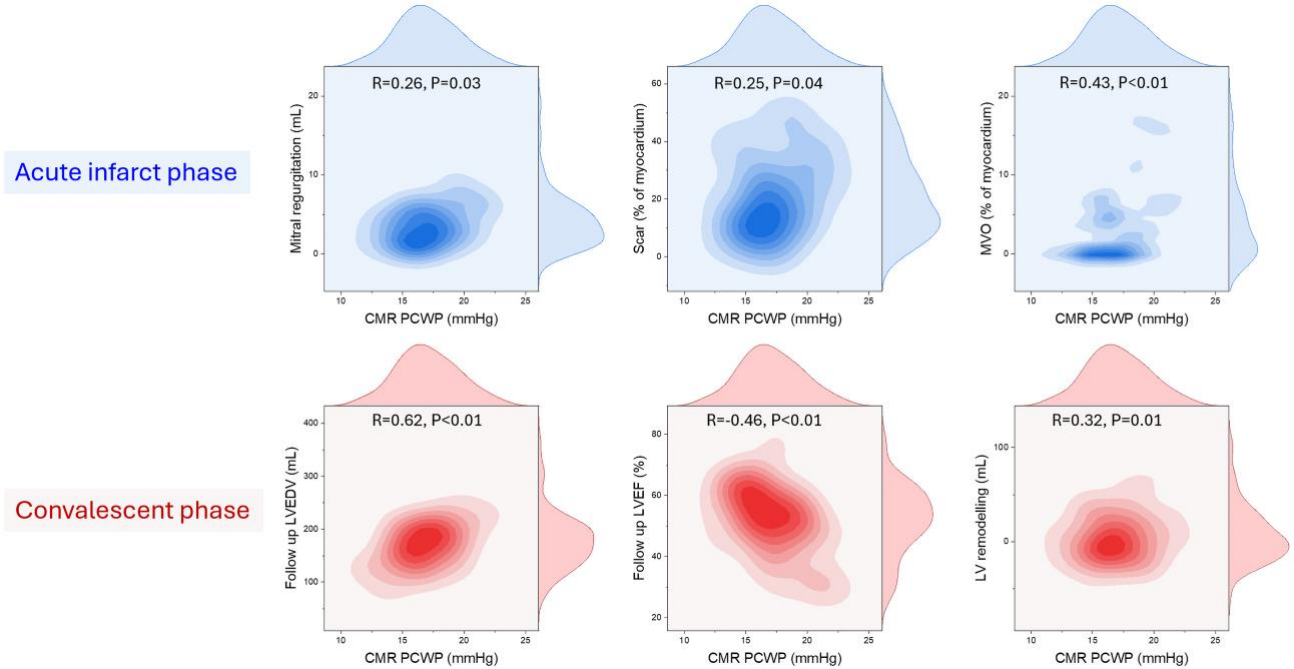
Associations between CMR-derived PCWP and key biomarkers in the acute infarct phase (top row) and structural cardiac changes in the convalescent phase (bottom row). In the acute phase, PCWP correlates positively with mitral regurgitation volume (*R* = 0.26, *P* = 0.03), scar burden (*R* = 0.25, *P* = 0.04), and myocardial microvascular obstruction (MVO, *R* = 0.43, *P* < 0.01). In the convalescent phase, CMR-derived PCWP is strongly associated with increased LV end-diastolic volume (EDV, *R* = 0.64, *P* < 0.01), reduced LV ejection fraction (EF, *R* = −0.46, *P* < 0.01), and greater LV remodelling (*R* = 0.32, *P* = 0.01). PCWP, pulmonary capillary wedge pressure; MVO, microvascular obstruction; LV, left ventricular; EDV, end-diastolic volume; EF, ejection fraction.

In the acute infarct phase, CMR-PCWP correlated positively with mitral regurgitation (*R* = 0.26, *P* = 0.03), scar size (*R* = 0.25, *P* = 0.04), and microvascular obstruction (*R* = 0.43, *P* < 0.01) (*Figure 3*). In the convalescent phase, CMR-PCWP correlated positively with LVEDV (*R* = 0.62, *P* < 0.01) and left ventricular remodelling (*R* = 0.32, *P* = 0.01), and negatively with LVEF (*R* = −0.46, *P* < 0.01).

### Prediction of convalescent LV function

**Figure 4:**
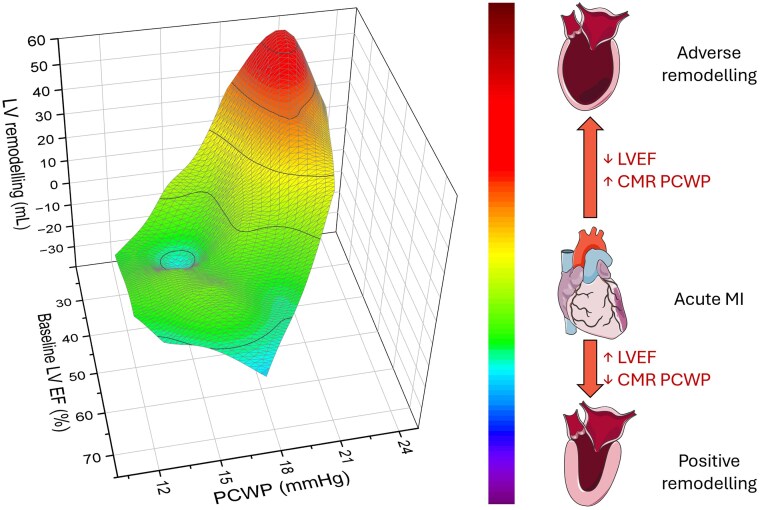
Three-dimensional surface-plot relationship between baseline LVEF, CMR-derived PCWP, and LV remodelling. The surface plot demonstrates that patients with higher CMR-derived PCWP and lower baseline LVEF exhibit the greatest LV remodelling (red regions), indicative of adverse structural changes. Conversely, lower CMR-derived PCWP and higher baseline LVEF are associated with minimal or even reverse positive remodelling (blue regions). Colour scale: The gradient from blue to red reflects the continuum of remodelling severity, with green and yellow regions representing intermediate degrees of LV volume change. LVEF, left ventricular ejection fraction; CMR, cardiovascular magnetic resonance; PCWP, pulmonary capillary wedge pressure; LV, left ventricular.

A stepwise multiple regression model revealed that baseline LVEF and baseline CMR PCWP were the only two independent predictors of follow-up LVEF (*Figure 4* and *Table 3*). Baseline LVEF showed a strong positive association (coefficient = 0.76, *P* < 0.0001), while baseline PCWP exhibited a modest but significant negative effect (coefficient = −1.01, *P* = 0.0036) (*Table 3*). Microvascular obstruction and scar burden were excluded from the final model due to a lack of independent contribution. Overall, the model explained 65% of the variance in follow-up LVEF (adjusted *R*^2^ = 0.6542, *P* < 0.0001), with a multiple correlation coefficient of 0.8151.

**Table 3:** Multiple regression analysis for predicting follow-up left ventricular ejection fraction (LVEF%)

Variable	Coefficient	Standard error	95% CI	*t*-Value	*P*-value
Constant	34.01	7.8	18.47 to 49.55	4.37	<0.0001
LV-EF baseline (%)	0.76	0.08	0.60 to 0.92	9.42	<0.0001
CMR PCWP (mmHg)	−1.01	0.33	−1.67 to −0.34	−3.02	0.0036
Microvascular obstruction (%)*					
Myocardial scar burden (%)*					
Age (years)*					
Sex (male/female)*					
*Removed from stepwise regression due to lack of independent association					
Adjusted *R*^2^	0.6542				
Multiple correlation coefficient	0.8151				
F-ratio	65.3089				
Significance Level	*P* < 0.0001				
Regression equation: LV-EF (%) = 34.01 + [0.76 × baseline LV-EF (%)] − [1.01 × baseline CMR PCWP (mmHg)]					

This analysis was conducted using stepwise multiple regression, where variables were included if *P* < 0.05 and removed if *P* > 0.1.

## Discussion

This study provides novel insights into the role of CMR-derived PCWP in the setting of STEMI. By leveraging a non-invasive, imaging-based estimation of LV filling pressure, we demonstrate that elevated CMR-derived PCWP is significantly associated with larger infarct size, greater MVO, and adverse LV remodelling following re-perfused STEMI. Importantly, high PCWP was associated with persistently reduced LVEF at follow-up, underscoring its potential as an early marker of ventricular dysfunction. Our findings reflect CMR-derived PCWP's untapped capacity as a risk stratification tool capable of identifying patients at heightened risk of adverse post-infarction remodelling and heart failure progression. The significant correlation identified between established markers of poor prognosis in acute myocardial infarction (MI) patients and CMR estimated PCWP, coupled with the feasibility of routine clinical implications, highlights CMR PCWP's potential to refine patient management and optimise targeted therapeutic interventions in post-STEMI care.

Elevated LVFP is an independent predictor of adverse outcomes in STEMI patients undergoing primary percutaneous coronary intervention (PCI). Studies have shown that higher LVFP is associated with increased mortality and adverse cardiovascular events. For instance, patients with LVEDP >18 mmHg had higher hazard ratios for death and re-infarction at both 30 days and 2 years compared to those with LVEDP ≤18 mmHg.^16^ Similarly, LVEDP >22 mmHg has been linked to higher rates of congestive heart failure, cardiogenic shock, and death at 90 days.^17^

Both our study and Marc et al. investigate predictors of LV remodelling post-STEMI, emphasizing early haemodynamic assessment for predicting significant clinical remodelling.^18^ Marc et al. utilized invasive coronary wedge pressure (CWP) before revascularization as a surrogate for microvascular obstruction, showing that elevated CWP (>38 mmHg) predicted greater LV dilation and reduced ejection fraction over 60 months. In contrast, we employed non-invasive CMR-derived PCWP within 48 h post-reperfusion, offering a safer, more widely applicable alternative. While both methods assess LV filling pressures, our approach circumvents procedural risks. However, our shorter 3-month follow-up may not capture long-term remodelling, and unlike Marc et al., who focused on high-risk anterior STEMI, our broader STEMI cohort enhances generalizability. These differences highlight the complementary value of both studies, with our findings reinforcing the potential of non-invasive CMR-derived PCWP for risk stratification.

Acute myocardial infarction is characterized by interstitial myocardial oedema that transiently augments LVM by roughly 10–20 g—equivalent to an 8–12% rise during the first post-ischaemic week.^19^ Although this oedema-related hypertrophy would tend to inflate the pressure estimate, the multivariate model is deliberately weighted towards the left atrial volume. The regression coefficient for LAV (0.07505 mmHg mL⁻^1^) exceeds that for LVM (0.05289 mmHg g⁻1) by ∼42%. Consequently, a 10-unit increment in LAV increases the predicted PCWP by 0.75 mmHg, whereas an identical (10-g) rise in oedematous LVM elevates the estimate by only 0.53 mmHg. This hierarchy of influence is physiologically appropriate as LAV integrates the chronic haemodynamic load on the ventricle. In contrast, the oedema-driven surge in LVM after AMI is transient and largely unrelated to steady-state filling pressures. The disproportionate weighting of the atrial term therefore mitigates the systematic error introduced by post-ischaemic myocardial swelling, ensuring that the CMR-derived surrogate remains primarily a reflection of true left-ventricular preload rather than of acute tissue water content.

In the study by Dregoesc et al., the authors investigated the relationship between echocardiographic parameters of MVO and LV remodelling over a 5-year follow-up in STEMI patients.^20^ Their findings indicated no significant association between echocardiographic markers of MVO and long-term LV remodelling. This contrasts with our study, where we employed CMR, the reference standard for MVO assessment. We established, for the first time, a direct correlation between MVO extent and elevated PCWP, providing mechanistic insights into the haemodynamic consequences of MVO post-STEMI. The discrepancy between the two studies may be attributed to differences in imaging modalities and methodologies. Echo, while widely accessible, may lack the sensitivity and specificity of CMR in detecting MVO, potentially leading to an underestimation of its prevalence and impact. Our use of CMR allowed for more precise quantification of MVO and its relationship with PCWP, thereby addressing previously debated topics regarding the impact of MVO on cardiac function. These findings underscore the importance of utilizing advanced imaging techniques like CMR to enhance risk stratification and management strategies in STEMI patients.

### Limitations

While our study provides valuable insights into the clinical significance of CMR-derived PCWP in STEMI patients, several limitations warrant consideration. First, the absence of invasive haemodynamic measurements precludes direct validation of the non-invasive PCWP estimates, potentially affecting the accuracy of our findings. However, CMR-derived PCWP has been validated against invasive measures in other clinical contexts, and its correlation with markers of adverse LV remodelling in our study supports its clinical relevance. Second, the study's observational design limits our ability to establish causality between elevated PCWP and adverse cardiac outcomes. Nonetheless, the physiological plausibility of our findings strengthens their clinical relevance. Following MI, increased preload leads to left atrium (LA) dilation as a compensatory response to elevated LV filling pressures,^21^ while increased LV mass reflects an after-loaded ventricle due to sustained pressure overload, impacting myocardial relaxation and compliance. The CMR-derived PCWP equation accounts for both LA volume and LV mass—two key determinants of LV filling pressures—reinforcing its mechanistic validity in this setting. However, our study does not account for myocardial oedema, which is typically elevated in the acute post-infarct phase and may transiently increase LV mass, potentially influencing PCWP estimation. Despite this, the limitation primarily affects the early phase and is less relevant to the predictive role of PCWP in long-term remodelling. Furthermore, the relatively small sample size and single-centre setting may restrict the generalizability of our results, though the rigorous imaging protocol enhances internal validity. Additionally, potential selection bias cannot be excluded, as patients with contraindications to CMR or those presenting with haemodynamic instability were not included. However, excluding such patients allowed for a more homogenous cohort, facilitating a clearer interpretation of CMR-derived PCWP in the context of LV remodelling. Lastly, we noted that our study cohort with >18 mmHg PCWP included mainly males. This is unlikely to represent sex-bias as the PCWP equation already factors in sex of the patient. This is likely because males tend to present with larger infarcts.^22,23^ Future studies incorporating invasive validation and larger and more diverse populations (with a female focus with higher PCWP), are necessary to confirm and extend our findings.

## Conclusions

CMR-derived PCWP may be a valuable clinical biomarker in STEMI, identifying patients at risk of adverse remodelling and LV dysfunction. Its integration into clinical practice may enhance risk stratification and guide targeted therapies.

## Supplementary Material

qyaf086_Supplementary_Data

## Data Availability

The data underlying this article will be shared at a reasonable request by the corresponding author.

## Declaration of Helsinki

This study complies with the Declaration of Helsinki and was approved by the National Research Ethics Service (16/YH/0352) in the UK.

## Lead author biography

Dr Pankaj Garg is an Associate Professor in Cardiovascular Medicine at the University of East Anglia, Norwich, UK and Honorary Consultant Cardiologist at Norfolk and Norwich University Hospitals. He graduated in Medicine with Honours he became a member of the Royal College of Physicians in 2007. After completing his BHF-funded PhD at Leeds (2017), he was awarded NIHR Clinical Lectureship programme at the University of Sheffield. In 2020, he was awarded Wellcome Trust Clinical Research Career Development Fellowship. His research focus is cardiovascular haemodynamics and Cardiac Magnetic Resonance Imaging. His ambition is to discover and advance a better understanding of the interface between cardiovascular flow and cardiac remodelling. He has received 20 national/international awards and has published >200 peer-reviewed papers.

## References

[1] Chenzbraun A, Keren A, Stern S. Doppler echocardiographic patterns of left ventricular filling in patients early after acute myocardial infarction. Am J Cardiol 1992;70:711–4. 1519519 10.1016/0002-9149(92)90546-b

[2] Kapur NK, O’Neill WW. Left ventricular end-diastolic pressure in acute myocardial infarction: a loaded target in need of unloading. Catheter Cardiovasc Interv 2019;93:910–1. 30953413 10.1002/ccd.28204

[3] Van Herck PL, Carlier SG, Claeys MJ, Haine SE, Gorissen P, Miljoen H et al Coronary microvascular dysfunction after myocardial infarction: increased coronary zero flow pressure both in the infarcted and in the remote myocardium is mainly related to left ventricular filling pressure. Heart Br Card Soc 2007;93:1231–7. 10.1136/hrt.2006.100818 PMC2000925 17395671

[4] Flaherty JT . Role of nitrates in acute myocardial infarction. Am J Cardiol 1992;70:73B–81B. 1529929 10.1016/0002-9149(92)90597-r

[5] Garg P, Gosling R, Swoboda P, Jones R, Rothman A, Wild JM et al Cardiac magnetic resonance identifies raised left ventricular filling pressure: prognostic implications. Eur Heart J 2022;43:2511–22. 35512290 10.1093/eurheartj/ehac207 PMC9259376

[6] Backhaus SJ, Schulz A, Lange T, Evertz R, Kowallick JT, Hasenfuß G et al Rest and exercise-stress estimated pulmonary capillary wedge pressure using real-time free-breathing cardiovascular magnetic resonance imaging. J Cardiovasc Magn Reson 2024;26:101032. 38431079 10.1016/j.jocmr.2024.101032 PMC10950869

[7] Thomson RJ, Grafton-Clarke C, Matthews G, Swoboda PP, Swift AJ, Frangi A et al Risk factors for raised left ventricular filling pressure by cardiovascular magnetic resonance: prognostic insights. ESC Heart Fail 2024;11:4148–59. 39132877 10.1002/ehf2.15011 PMC11631267

[8] Garg P, van der Geest RJ, Swoboda PP, Crandon S, Fent GJ, Foley JRJ et al Left ventricular thrombus formation in myocardial infarction is associated with altered left ventricular blood flow energetics. Eur Heart J Cardiovasc Imaging 2019;20:108–17. 30137274 10.1093/ehjci/jey121 PMC6302263

[9] Demirkiran A, Hassell MECJ, Garg P, Elbaz MSM, Delewi R, Greenwood JP et al Left ventricular four-dimensional blood flow distribution, energetics, and vorticity in chronic myocardial infarction patients with/without left ventricular thrombus. Eur J Radiol 2022;150:110233. 35278980 10.1016/j.ejrad.2022.110233

[10] Ibanez B, James S, Agewall S, Antunes MJ, Bucciarelli-Ducci C, Bueno H et al 2017 ESC Guidelines for the management of acute myocardial infarction in patients presenting with ST-segment elevation: the Task Force for the management of acute myocardial infarction in patients presenting with ST-segment elevation of the European Society of Cardiology (ESC). Eur Heart J 2018;39:119–77. 28886621 10.1093/eurheartj/ehx393

[11] Garg P, Grafton-Clarke C, Matthews G, Swoboda P, Zhong L, Aung N et al Sex-specific cardiac magnetic resonance pulmonary capillary wedge pressure. Eur Heart J Open 2024;4:oeae038. 38751456 10.1093/ehjopen/oeae038 PMC11095051

[12] Haslak F, Yildiz M, Adrovic A, Sahin S, Koker O, Aliyeva A et al Management of childhood-onset autoinflammatory diseases during the COVID-19 pandemic. Rheumatol Int 2020;40:1423–31. 32661928 10.1007/s00296-020-04645-x PMC7355083

[13] Sugimoto T, Dohi K, Tanabe M, Watanabe K, Sugiura E, Nakamori S et al Echocardiographic estimation of pulmonary capillary wedge pressure using the combination of diastolic annular and mitral inflow velocities. J Echocardiogr 2013;11:1–8. 23555178 10.1007/s12574-012-0142-0 PMC3611026

[14] Guzzetti S, La Rovere MT, Pinna GD, Maestri R, Borroni E, Porta A et al Different spectral components of 24 h heart rate variability are related to different modes of death in chronic heart failure. Eur Heart J 2005;26:357–62. 15618038 10.1093/eurheartj/ehi067

[15] Aalders M, Kok W. Comparison of hemodynamic factors predicting prognosis in heart failure: a systematic review. J Clin Med 2019;8:1757. 31652650 10.3390/jcm8101757 PMC6832156

[16] Planer D, Mehran R, Witzenbichler B, Guagliumi G, Peruga JZ, Brodie BR et al Prognostic utility of left ventricular end-diastolic pressure in patients with ST-segment elevation myocardial infarction undergoing primary percutaneous coronary intervention. Am J Cardiol 2011;108:1068–74. 21798494 10.1016/j.amjcard.2011.06.007

[17] Bagai A, Armstrong PW, Stebbins A, Mahaffey KW, Hochman JS, Weaver WD et al Prognostic implications of left ventricular end-diastolic pressure during primary percutaneous coronary intervention for ST-segment elevation myocardial infarction: findings from the Assessment of Pexelizumab in Acute Myocardial Infarction study. Am Heart J 2013;166:913–9. 24176448 10.1016/j.ahj.2013.08.006

[18] Marc MC, Iancu AC, Ober CD, Homorodean C, Bãlãnescu Ş, Sitar AV et al Pre-revascularization coronary wedge pressure as marker of adverse long-term left ventricular remodelling in patients with acute ST-segment elevation myocardial infarction. Sci Rep 2018;8:1897. 29382891 10.1038/s41598-018-20276-6 PMC5789971

[19] Zagrosek A, Wassmuth R, Abdel-Aty H, Rudolph A, Dietz R, Schulz-Menger J. Relation between myocardial edema and myocardial mass during the acute and convalescent phase of myocarditis—a CMR study. J Cardiovasc Magn Reson 2008;10:19. 18447954 10.1186/1532-429X-10-19 PMC2396625

[20] Dregoesc MI, Iancu AC, Ober CD, Homorodean C, Bãlãnescu Ş, Bolboacã S. In ST-segment elevation myocardial infarction, the echocardiographic parameters of microvascular obstruction are not associated with left ventricular remodeling at five years of follow-up. Echocardiography 2019;36:1103–9. 31116460 10.1111/echo.14371

[21] Saklecha A, Kapoor A, Sahu A, Khanna R, Kumar S, Garg N et al Is indexed left atrial volume (LAVi) in Indian patients with acute coronary syndrome (ACS) undergoing revascularization a predictor of cardiovascular outcomes? Ann Card Anaesth 2022;25:19–25. 35075016 10.4103/aca.ACA_129_20 PMC8865354

[22] De Luca G, Parodi G, Sciagrà R, Bellandi B, Verdoia M, Vergara R et al Relation of gender to infarct size in patients with ST-segment elevation myocardial infarction undergoing primary angioplasty. Am J Cardiol 2013;111:936–40. 23332594 10.1016/j.amjcard.2012.12.011

[23] Krefting J, Graesser C, Novacek S, Voll F, Moggio A, Krueger N et al Sex-specific outcomes in myocardial infarction: a dual-cohort analysis using clinical and real-world data. Clin Res Cardiol 2025. 10.1007/s00392-025-02627-2 PMC12708805 40111442

