# Supplementary material for: Haemodynamic implications of cardiovascular magnetic resonance pulmonary capillary wedge pressure in acute myocardial infarction
Source: Eur Heart J Imaging Methods Pract. 2025 Jul 25;3(2):qyaf086. doi: 10.1093/ehjimp/qyaf086 (PMC12311366; doi:10.1093/ehjimp/qyaf086)
Supplement: qyaf086_Supplementary_Data [file qyaf086_supplementary_data.docx]

# Supplementary Information Document

**Supplementary Table 1**: Kolmogorov-Smirnov Test for assessing normal distribution stratified according to acute and convalescent phase CMR, and CMR-derived PCWP (PCWP ≤18 mmHg vs >18 mmHg). The table represents the p-values for the test, where a statistically significant result (p<0.05) rejects the null hypothesis, indicating deviation of data from normal distribution.

|  | **CMR PCWP ≤ 18 mmHg** | **CMR PCWP > 18 mmHg** |
| --- | --- | --- |
|  | Normal Distribution | Normal Distribution |
| **Acute phase CMR study (within 48 hours)** |  | |
| Left Ventricular End-Diastolic Volume (mL) | 0.30 | 0.06 |
| Left Ventricular End-Systolic Volume (mL) | 0.90 | 0.20 |
| Left Ventricular Stroke Volume (mL) | 0.42 | 0.61 |
| Left Ventricular Ejection Fraction (%) | 0.77 | 0.06 |
| Left Ventricular End-Diastolic Mass (g) | 0.53 | **0.03** |
| Myocardial Scar Percentage (%) | **0.03** | 0.54 |
| Microvascular Obstruction (%) | **<0.0001** | 0.20 |
| Left Atrial volume (mL) | 0.81 | 0.26 |
| **Convalescent phase CMR study (at 3 months)** |  | |
| Mitral Regurgitation (mL) | **0.00** | 0.21 |
| Left Ventricular End-Diastolic Volume (mL) | 0.10 | 0.34 |
| Left Ventricular End-Systolic Volume (mL) | 0.58 | 0.07 |
| Left Ventricular Stroke Volume (mL) | 0.85 | 0.45 |
| Left Ventricular Ejection Fraction (%) | 0.49 | 0.25 |
| Left Atrial volume (mL) | 0.73 | 0.63 |

**Supplementary Table 2**: Sensitivity analysis conducted on the non-normally distributed variables through performing an Independent Samples Mann-Whitney U Test comparing their distribution across both CMR-derived PCWP groups (PCWP ≤18 mmHg vs >18 mmHg). The statistical significance level was set at p < 0.05. Results were compared to a parametric, Independent Samples T-test.

|  | **Mann-Whitney U Test p-value** | **Independent Samples T-test p-value** |
| --- | --- | --- |
| Left Ventricular End-Diastolic Volume (mL) | 0.001 | 0.0001 |
| Myocardial Scar Percentage (%) | 0.001 | 0.0008 |
| Microvascular obstruction (%) | <0.0001 | <0.0001 |
| Mitral Regurgitation (mL) | 0.003 | 0.0084 |
